# Blood‐based biomarkers of systemic inflammation, metabolic function, and vascular injury are elevated in patients with rapid progressive neurodegenerative disease

**DOI:** 10.1002/alz.090378

**Published:** 2025-01-09

**Authors:** Lindsey A Kuchenbecker, Philip W Tipton, Matthew R Brier, Nihal Satyadev, Yoav D Piura, S Richard Dunham, Sid E. O'Bryant, Neill R Graff‐Radford, Gregory S Day

**Affiliations:** ^1^ Mayo Clinic, Jacksonville, FL USA; ^2^ Washington University School of Medicine, Saint Louis, MO USA; ^3^ University of North Texas Health Science Center, Fort Worth, TX USA; ^4^ Department of Neurology, Mayo Clinic, Jacksonville, FL USA

## Abstract

**Background:**

Patients with rapid progressive Alzheimer disease and related dementias (rpAD/ADRD) develop dementia within 1 year or incapacitation within 2 years of symptom onset. We previously showed that selected CSF biomarkers of neuronal injury (NfL, VILIP‐1), AD neuropathology (p‐tau181), and neuroinflammation (GFAP, MCP‐1, sTREM2) measured at presentation associated with etiologic diagnoses and reliably differentiated patients with treatment‐responsive causes of rapid progressive dementia. However, no differences were identified between CSF biomarkers in patients with rapid and typical progressive forms of AD/ADRD, leaving key questions unanswered concerning the mechanisms that drive rpAD/ADRD. Blood‐based biomarkers may inform the systemic factors that contribute to rpAD/ADRD, including the systemic inflammation, metabolic function, and vascular injury.

**Methods:**

Biomarkers of systemic inflammation (Eotaxin‐3, IFN‐γ, IL‐1b, IL‐2, IL‐4, IL‐6, IL‐8, IL‐10, IL‐12p70, IL‐13, TARC, TNF‐α), metabolic function (A2M, B2M, FABP‐3, FVII, I‐309, IL‐18, PPY, TNC, TPO), and vascular injury (CRP, SAA, sICAM‐1, sVCAM‐1) were measured in plasma obtained at presentation from patients with rpAD/ADRD (n=29) and age‐ and sex‐ similar participants with typical progressive AD/ADRD (n=39) enrolled in prospective studies of memory and aging at Mayo Clinic in Florida. Biomarker measures were completed at the University of North Texas Institute for Translational Research using Meso Scale Discovery multiplexed assays. Biomarker concentrations were compared between groups using univariate analyses; potential predictors (p<0.05) were further evaluated via step‐wise multivariate logistic regression.

**Results:**

Markers of vascular injury (SAA, CRP, sICAM‐1), metabolic function (I‐309, TPO), and systemic inflammation (Eotaxin‐3, TARC, TNF‐α) were increased in patients with rapid versus typical progressive AD/ADRD (p<0.05). Eotaxin‐3, I‐309, and SAA were independently associated with rpAD/ADRD in multivariable model after controlling for age and sex. The overall model correctly characterized 86.8% of patients, with an area under the curve of 0.864 (95%CI: 0.777‐0.950; p<0.001).

**Conclusion:**

Blood‐based biomarkers of systemic inflammation, metabolic function, and vascular injury were elevated at presentation in patients with rapid vs typical AD/ADRD. These findings may provide additional insights into the mechanisms that drive rpAD/ADRD. Longitudinal measures from larger cohorts are needed to decipher the relationship between candidate biomarkers and rpAD/ADRD.

